# Integrated In Silico – In Vitro Study Investigating Dipeptides as Chorismate Synthase Modulators: Spotlight on Its Mechanism of Action

**DOI:** 10.1002/gch2.202500316

**Published:** 2025-10-30

**Authors:** Lorenzo Pedroni, Katharina Fuchs, Gianni Galaverna, Peter Macheroux, Luca Dellafiora

**Affiliations:** ^1^ Department of Food and Drug University of Parma Parma Italy; ^2^ Institute of Biochemistry Graz University of Technology Graz Austria

**Keywords:** bioactive peptides, *Campylobacter jejuni*, chorismate synthase, food safety, hybrid approach

## Abstract

*Campylobacter jejuni* is a widespread foodborne pathogen causing campylobacteriosis, a disease leading to diarrhea, fever, and gastroenteritis, able to adapt to many niches. Here, we present a hybrid in silico/in vitro study investigating the modulation of *C. jejuni* chorismate synthase by peptides. This enzyme belongs to the shikimate pathway, and it is an interesting target for selective growth modulation, being crucial for bacteria but not present in animals. To account for the identification of “natural” modulators, a library of 400 dipeptides is screened in silico through docking and molecular dynamics simulations to identify possible inhibiting sequences. The dipeptide glutamate‐aspartate (ED) stood out, emulating the pharmacophoric fingerprint and interaction of the enzyme's natural substrate. Serendipitously, in vitro trials revealed ED as an activity enhancer. Considering the growth of *C. jejuni* in protein‐rich matrices, this outlined a possibly relevant matrix‐dependent effect worthy of dedicated investigations. The underpinning mechanisms are computationally investigated, describing possible ED‐dependent effects on substrate/product turnover and enzyme structural stability. This study deepened the understanding of chorismate synthase and opened new directions in designing food‐grade peptide‐based modulators. This may provide ground to improve controlling bacterial growth in diverse contexts, including food safety and environmental/agricultural hygiene.

## Graphical abstract

This study integrates computational and experimental approaches to explore how short dipeptides modulate the activity of chorismate synthase. Unexpectedly, the GluAsp dipeptide enhanced enzyme activity by promoting a transient surface channel near the active site, possibly revealing a new structural feature relevant for microbial metabolism and modulator design.

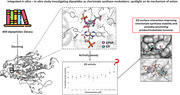

## 1 Introduction

*Campylobacter jejuni* (CJ) is a widespread bacterium in many food‐related niches [1] and is among the main causes of foodborne illnesses worldwide, as per the World Health Organization [2]. It is usually transmitted through contaminated and undercooked poultry, foods cross‐contaminated with raw poultry products, or from raw and unpasteurized milk; however, there can be other ways of infection, such as seafood and vegetables [3, 4, 5, 6, 7]. It is a gram‐negative bacterium causing campylobacteriosis, an infection including gastroenteritis, fever, and diarrhea [8]. There are more than 1 million cases registered every year, causing a significant burden of disease [9]. Apart from its typical symptoms, this illness could lead to significant complications such as the Guillain–Barrè syndrome, causing numbness, paralysis, and eventually death [10]. CJ preferentially grows in microaerophilic conditions, and it is usually inhibited by refrigeration temperatures [11]. However, it can form biofilms, which enhance its resistance and persistence; additionally, recent studies have identified cold‐resistant CJ strains capable of surviving under aerobic conditions, posing more challenges from a food safety standpoint [12, 13, 14].

Prokaryotes, including CJ, evolved a specific biosynthetic route to produce aromatic amino acids, the shikimate pathway, which is fundamental for their survival [15]. This pathway is characterized by seven steps that, starting from phosphoenolpyruvate, lead to the production of chorismate, the last precursor of folates and aromatic amino acids [16]. This pathway is a crucial pathway for bacterial growth and a promising target for developing strategies modulating bacterial growth with no expected effects on animals, as they lack this pathway [17]. Indeed, researchers have already tried to target it by attempting to develop broad‐spectrum antibacterial compounds due to the high conservation within the bacterial phyla [18, 19, 20]. At the same time, the identification of food‐grade compounds that target the enzymes of that pathway is highly desirable to take steps toward the implementation of safe and sustainable containment/modulating strategies through the food chain.

In this context, we concentrated our analysis on dipeptides, which are inherently food‐grade and safe molecules, to identify sequences that may interact with and modulate CJ chorismate synthase (CS). CS is the final enzyme in the seven‐step shikimate pathway and catalyses the 1,4‐trans elimination of the phosphate group from its natural substrate 5‐enolpyruvylshikimate‐3‐phosphate (EPSP) through a reaction exploiting flavin mononucleotide (FMN) as a cofactor [21]. Furthermore, its crystallographic structure is now accessible in the Protein Data Bank (https://www.rcsb.org/) [22], facilitating the development of 3D in silico modelling studies. This may enable comprehensive in silico screenings to identify CS‐modulating peptides, since short acidic peptides may resemble certain chemical motifs of EPSP, potentially allowing them to competitively bind with the natural substrate. Therefore, we applied an in silico pipeline based on molecular docking and dynamics simulations since it already succeeded in discovering bioactive compounds, including peptides [23, 24, 25, 26]. We built a library containing all 400 possible dipeptides given by the combination of all 20 proteinogenic amino acids. This was used as input for the subsequent molecular docking simulation, while the most promising candidates were also investigated via molecular dynamics simulations to refine predictions in agreement with previous studies [24, 25]. The considered best hit (the dipeptide glutamate‐aspartate; ED) and worst hit (the dipeptide valine‐alanine; VA) were then analyzed through in vitro CS activity assay as per previous investigations [27, 28]. The choice of seeking bioactive peptides, and dipeptides specifically, was in line with previous research proving the intrinsic antimicrobial properties of certain sequences and their possible multi‐purpose use as food/feed additives [29, 30, 31, 32]. Moreover, investigating the behavior of dipeptides in their interaction with CJ CS is fundamental to a better understanding of the impact that nutritional sources may have on CJ, since it is commonly found within protein‐rich matrices [11]. These insights could help improve the understanding of crucial chemical aspects related to food safety and production, and to design more effective chemical‐based strategies to control bacterial growth in food matrices.

## 2 Materials and Methods

### 2.1 Data Source

The 3D structure of CJ CS is stored on the Protein Data Bank (PDB; https://www.rcsb.org/) [33] under the PDB ID 1SQ1 [22]. However, since the structure is not fully resolved, it was modelled through ColabFold (v 1.5.5) [34], a publicly available notebook implemented with AlphaFold2 and AlphaFold2‐multimer [35, 36], in agreement with previous studies [37]. Primary sequences of *C. jejuni* CS and its *Paracoccidioides brasiliensis* orthologue, the latter used as a valid model system for CS according to recent papers [28], were collected from UniProt (https://www.uniprot.org; UniProt ID Q9PM41 and A0A1D2J6D4, respectively) [38] and given as input to ColabFold along with 1SQ1, the latter used as a custom template. Once a reliable model was obtained, the cofactor FMN was added and optimized within CS's binding site through AlphaFill (https://alphafill.eu/) [39], setting default parameters.

The 3D structure of gallion (CID 135406014), a known CS inhibitor exhibiting an IC_50_ of 10 ± 1 µm, and EPSP (439463), i.e., the native substrate, were downloaded in the SDF format from PubChem (https://pubchem.ncbi.nlm.nih.gov/) [40]. They were converted to the MOL2 format through Open Babel (v. 3.1.1) [41] before proceeding with the next steps.

### 2.2 Construction of the Dipeptides Library

The library of dipeptides analyzed in this study was generated using an *ad hoc* Python script executed through PyMol (v.2.3), in line with a previous study [37]. All possible combinations of the 20 proteinogenic amino acids were created, obtaining 400 dipeptides in the MOL2 format. These were protonated at pH 7, each with a deprotonated C‐terminus and protonated N‐terminus, as per physiological conditions.

### 2.3 Molecular Docking Simulations

Molecular docking simulations were performed through the software GOLD (Genetic Optimization for Ligand Docking; v. 2022) [42]. A semi‐flexible docking approach was applied, allowing only the protein's polar hydrogens to be free to rotate while keeping the ligands fully flexible. The binding site was set as a 10 Å sphere centered within the CS's active site, keeping the FMN cofactor. 10 poses per ligand were generated and scored using the internal PLPScore score function (the higher the score, the more likely the predicted architecture of binding, as per manufacturer declarations; https://www.ccdc.cam.ac.uk/), in line with previous studies [37]. The same procedure, but changing the centroid, was repeated to dock the peptides within the identified surface's groove.

Last, the stability of only those ligands that were tested with molecular dynamics simulations was further evaluated using the PRODIGY webserver [43] employing default parameters in accordance with previous studies [44].

### 2.4 Pharmacophoric Analysis of CS Pocket

A pharmacophoric analysis of the CJ CS binding site was conducted using the IsoMif algorithm [45]. This was done to obtain precise insights into the CS's binding pocket chemical fingerprint, specifically focusing on regions capable of accommodating negatively charged groups. Default parameters were used while setting the maximum distance value between the grid and residue atoms at 3 Å with a grid resolution of 1 Å.

### 2.5 Molecular Dynamics Simulations

Molecular dynamics (MD) simulations were executed using GROMACS (v. 2021.4) [46] to verify the evolution over time of the complexes obtained from the molecular docking simulations. Of note, the only tested complexes were four, namely: CS‐EPSP, CS‐gallion, and CS with 2 different dipeptides, i.e., ED and VA.

Both the protein and peptides were parametrized with the CHARMM27 all‐atom forcefield [47] while the non‐protein ligands (i.e., gallion and EPSP) were on the SwissParam webserver (https://old.swissparam.ch/) [48]. Each system was placed in an octahedral box, solvated with SPC/E waters, and neutralized by adding Na^+^ and Cl^−^ ions. Every system, each consisting of ∼80 000 atoms, was then energetically minimized to avoid steric clashes or to correct improper geometries. This was achieved using a steepest descent algorithm with a maximum of 5000 optimization steps. Each minimized system underwent isothermal (300 K, 2 ps coupling time) and isobaric (1 bar, 2 ps coupling time) simulations for 100 ps to allow the system to reach equilibrium. Once these steps are completed, 30 ns long MD simulations were run.

The same procedure to test the action of ED and VA bound to the identified surface's groove on the Pb CS. Moreover, an additional MD simulation was run on the Pb CS without any bound peptide. For this investigation, MD simulations were run for 100 ns.

### 2.6 Chorismate Synthase Activity's Assay

To evaluate the modulatory effects of dipeptides (i.e., ED and VA) on CS activity, we employed a coupled assay using the well‐known CS inhibitor gallion as a positive control. The activity assay was performed using recombinant *P. brasiliensis* CS due to its high production yield and the availability of a validated fluorescence‐based coupled assay [27, 28]. This assay enables the forward coupling of the CS (from *P. brasiliensis*) reaction with anthranilate synthase, which processes chorismate, resulting in the production of anthranilate, a stable compound with fluorescence properties suitable for spectrofluorometric detection [27, 28]. All the chemicals and peptides were purchased from Merck (peptides at 95% purity), handled and stored prior to usage according to the supplier's specifications.

Reaction mixtures were prepared in 50 mm MOPS (pH 7.5) containing the following components at final concentrations: 3 mm MgSO_4_, 7.5 mm L‐Gln, 22.5 mm (NH_4_)_2_SO_4_, 1 mm dithiothreitol (DTT), 10 µm FMN, 80 µm EPSP (i.e., the CS substrate), 4 µm CS, 20 µm anthranilate synthase. Various concentrations of gallion and tested dipeptides ED and VA (0.1–500 µm) were added to assess their modulatory effects.

The reaction mixtures were then pipetted into a 96‐well plate in triplicate. The reaction was initiated by adding 500 µm NADPH and monitored spectrofluorometrically at 37°C as follows: anthranilate formation was monitored by measuring the fluorescence emission at 390 nm (λ_ex_ = 340 nm) using a Spectramax Gemini XS Microplate Spectrofluorometer (Molecular Devices, CA, USA). Fluorescence measurements were recorded every 12 s for 5 min to capture the initial linear phase of the reaction. The increase in fluorescence intensity directly correlates with anthranilate production, which is stoichiometrically related to chorismate formation by the CS enzyme. Initial reaction velocities were determined from the linear portion of the fluorescence versus time curves. The slopes of the initial velocities were determined and plotted as a function of the log of the corresponding tested compounds' concentration. All measurements were performed in triplicate (*n* = 3). Data are expressed as the mean ± standard error mean (SEM). Statistical analysis was performed using IBM SPSS Statistics for Windows, version 29 (IBM Corp., Armonk, NY). The data were analyzed by one‐way ANOVA (α = 0.05), followed by *post hoc* Fisher's LSD test (α = 0.05) to identify significant differences between treatments.

## 3 Results and Discussion

### 3.1 3D Model Validation

**FIGURE 1:**
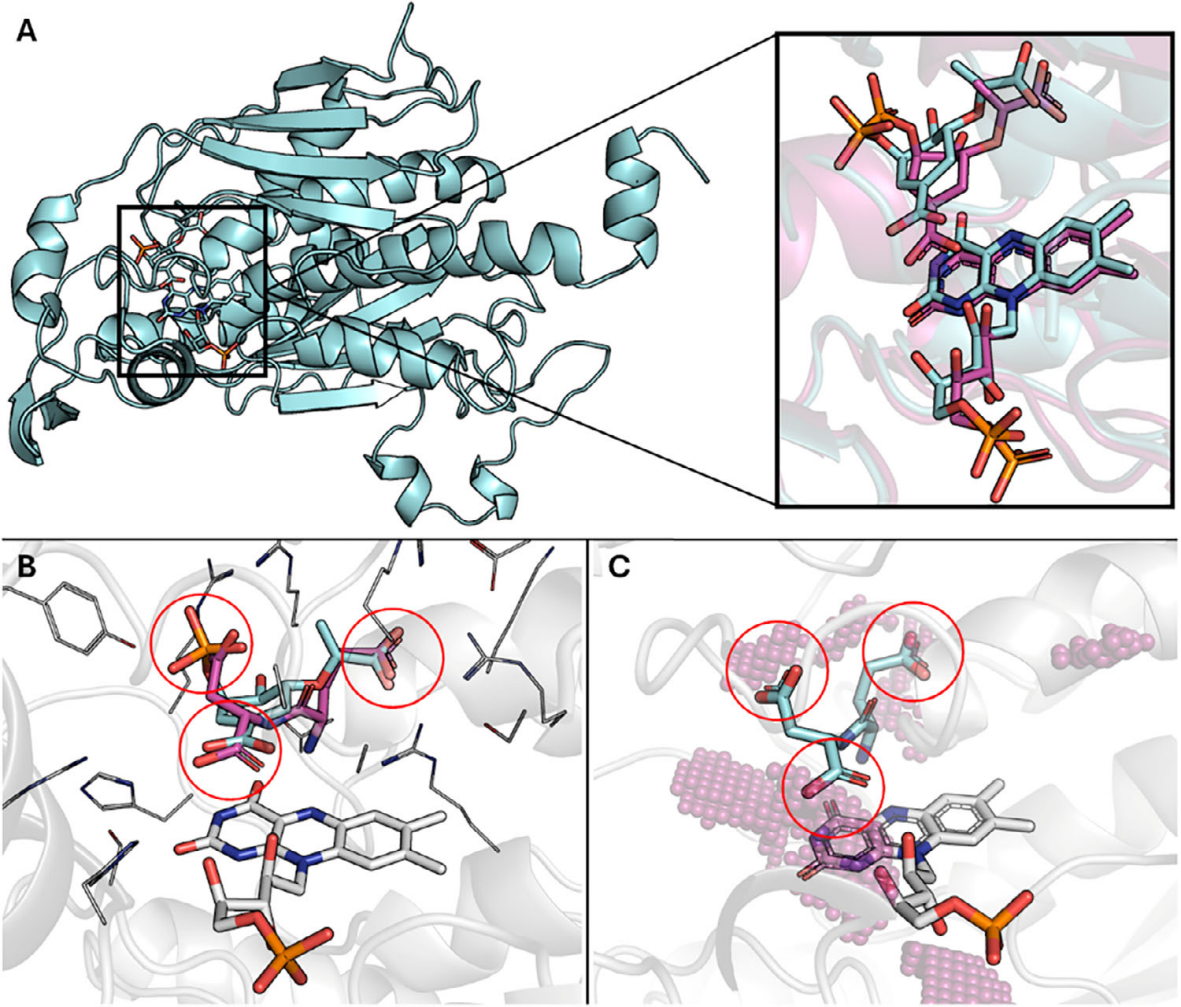
A) On the left, the 3D structure of the *C. jejuni* CS monomer is represented as a cyan cartoon while FMN and EPSP are shown as cyan sticks. On the right, close‐up on the binding site with FMN and EPSP within *C. jejuni* CS (cyan) compared to the co‐crystallized ligands within *S. pneuomoniae* CS (magenta; PDB ID 1QXO [36], ∼1.5 Å RMSD). (B, C) Focus on the CS binding site with the protein represented as a white transparent cartoon, residues as white lines, FMN as white sticks. Negatively charged moieties are embedded by red circles. B) Superimposition of EPSP (magenta) and ED (cyan). C) Regions accepting negatively charged groups are represented as a magenta sphere, while ED is represented as cyan sticks.

CJ CS was modelled through ColabFold (v 1.5.5) [34] (details in Section 2.1), obtaining a reliable structure with a pLDDT score – a measure ranging from 0 to 100 giving the per‐residue local confidence of the generated model – higher than 90, indicating well‐predicted backbone and side chains. Since CS is a homotetramer with four identical binding sites, all subsequent analysis was performed on a single monomer, also to reduce the computational load. Subsequently, the architecture of binding of the FMN cofactor, added through AlphaFill (https://alphafill.eu/) [39] within the CS binding site, was comparable to the *Streptococcus pneumoniae* CS crystallographic structure (PDB ID 1QXO [49]), which was the only structure with co‐crystallized cofactor and EPSP at the time of analysis (Figure 1A).

Once the reliability of the predicted CS‐FMN complex was assessed (Figure 1A) we proceeded with molecular docking simulations. The accuracy of the applied docking protocol, prior to using it for gallion (the inhibitor reference used here [28]) and the whole dipeptide library, was assessed on EPSP. Specifically, the architecture of binding of EPSP (PLPScore 70.03 units, −9 kcal/mol) obtained after the docking procedure was almost superimposed to the one co‐crystallized within *S. pneumoniae* CS crystallographic structure (PDB ID 1QXO [49], ∼1.5 Å RMSD), confirming both the model and procedural reliability.

### 3.2 Dipeptides Library Docking‐Based Screening

**FIGURE 2:**
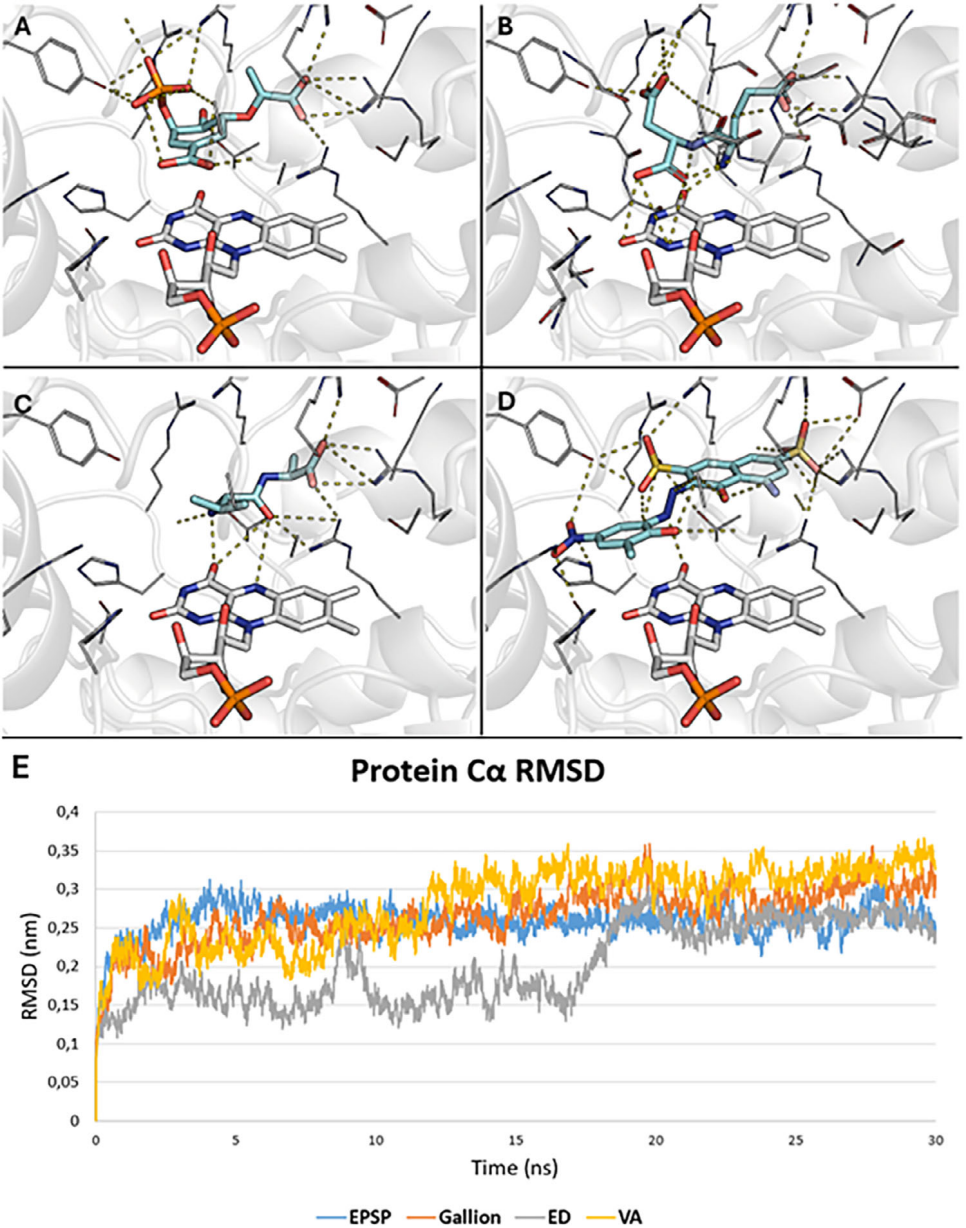
A–D) Docking poses of the four chosen compounds for MD simulation and in vitro testing. Ligands, namely EPSP (A), ED (B), VA (C), and gallion (D), are represented as cyan sticks. Protein is represented as a white transparent cartoon, while residues around ligands are shown as white lines. Polar interactions are represented as dashed lines. (E) CS Cα RMSD computed over the 30 ns long MD simulations. EPSP is reported in blue (average 0.259 ± 0.021 nm), gallion in orange (average 0.267 ± 0,0369 nm), ED in grey (average 0.202 ± 0.051 nm), and VA in yellow (average 0.281 ± 0.050 nm).

The dipeptides library (400 peptides in total) was constructed using an *ad hoc* Python script implemented with PyMol (v. 2.3), in line with a previous study [37]. Specifically, the script takes as input the structure of a C‐terminus proteinogenic amino acid and iteratively bonds an N‐terminus proteinogenic amino acid to it, maintaining a proper protonation state under physiological conditions (pH 7). Eventually, it stores the 3D coordinates of the generated dipeptide in the MOL2 format, a proper format for the docking procedure performed with GOLD. This 400 dipeptide library was screened exploiting the docking protocol validated using EPSP (see Section 3.1). Peptides were selected as the chemical space to identify CS modulators since they are expected to be generally safe and have already proved antibacterial properties [29, 30, 50]. However, only dipeptides were considered due to the limited size of the CS binding site, which likely prevents the interaction of longer peptides, and thus, only linear conformations could have been considered. The obtained docking scores were all positive, roughly ranging from 50 to 95 PLPScore units, indicating that all the dipeptides could theoretically interact with the CS binding site (the higher the score, the higher the physico‐chemical ligand‐pocket match; as per manufacturer declaration, https://www.ccdc.cam.ac.uk). However, an expert visual inspection of the top 10%‐based on the docking scores (Table S1)‐of obtained poses was performed through PyMol (v. 2.3) to seek the best possible hit to analyze through MD simulations and to eventually test in vitro. In more detail, focusing on the dipeptides binding architecture, it was noticed that certain short acidic peptides, as expected, might convincingly emulate some of the chemical motifs of the CS substrate, suggesting a possible role as competitive inhibitors. Among the 400 tested, ED (PLPScore 84.44 units, −6 kcal/mol) was selected as the most promising. Although other dipeptides exhibited higher PLPScores (see Table S1), their architecture of binding showed a lower correspondence with the EPSP (PLPScore 70.03 units, −9 kcal/mol) and CS catalytic pocket pharmacophoric fingerprint. Therefore, ED was selected as the top candidate since it was the one best emulating the pattern of interaction of EPSP, the natural CS substrate (Figure 1B,C). Indeed, as shown in Figure 1B,C, the Glu (E) α‐carboxylate moiety was superimposed on the EPSP's carboxylate moiety next to FMN. Similarly, the Glu γ‐carboxylate moiety occupied the regions corresponding to the other EPSP's carboxylate group, while the Asp (D) β‐carboxylate moiety occupied the region of the EPSP's phosphate group. Additionally, the pharmacophoric analysis of the CS binding pocket with IsoMif [45], performed to find regions likely to accommodate negatively charged groups, confirmed the goodness of such interaction with respect to the pharmacophoric fingerprint of the pocket (Figure 1B,C). On the other hand, among the peptides tested, we included VA (PLPScore 58.29 units, −6 kcal/mol) as a negative hit to test both with MD simulation and in vitro assay. This dipeptide is significantly different from ED due to its more pronounced hydrophobic nature. Specifically, it lacks negatively charged side chains, and it is engaged in a different pattern of interaction than ED and EPSP, which were deemed likely to result in a less favored interaction with the enzyme (Figure 2; Figure S1).

Finally, gallion (PLPScore 43.75 units, −8 kcal/mol), a recently described CS inhibitor [28], like others already characterized [27, 28], was calculated as a positive control. The pattern of interaction was comparable to, and even denser than, those of ED and EPSP (Figure 2; Figure S1).

### 3.3 MD Simulations and In Vitro Assay

#### 3.3.1 MD Simulations

Only four of the complexes obtained from the docking simulations, namely CS‐EPSP, CS‐gallion, CS‐ED, and CS‐VA, served as input for 30 ns long MD simulations. As shown in Figure 2, with respect to the overall protein stability – monitored via root mean squared deviation (RMSD) trends analysis – all the complexes were comparable from the second half of the MD simulation. VA was considered the worst based on its higher RMSD average value, reflecting its worse geometrical stability. Of note, the analysis of the radius of gyration (Rg) confirmed the better stability of CS when complexed with either ED or EPSP, while complexes with VA and gallion appeared more flexible and less compact (Figure S2). Moreover, the calculation of all‐atom RMSDs between the initial and the final snapshot from the MD simulations further corroborated the stability of ED and EPSP, with an RMSD between the two frames of 1.99 and 1.69 Å, respectively. Conversely, gallion and VA were less stable with an RMSD of 3.89 and 2.71 Å, respectively (Figure S2).

Subsequently, an H‐bond analysis monitoring the evolution of H‐bond numbers over time was conducted on all four tested complexes (Figure S3). Specifically, it was observed that CS‐EPSP maintained a good amount of H‐bonds, more than 9 on average (9.48 ± 1.01), over the 30 ns MD simulation. Moving to gallion, it kept a similar but slightly lower amount of H‐bonds during the simulation (9.20 ± 1.14) while ED was the best, maintaining more than 10 H‐bonds on average (10.65 ± 1.30). On the other hand, VA was the worst, as expected from the docking pose, keeping less than 7 H‐bonds on average (6.57 ± 1.11) due to the lack of side chains useful for interacting with the polar environment of the pocket. These data suggested that, in line with its similarity with EPSP and gallion, ED could be a promising competitive CS inhibitor, while VA should not obtain any result of this kind.

#### 3.3.2 Activity Assay

**FIGURE 3:**
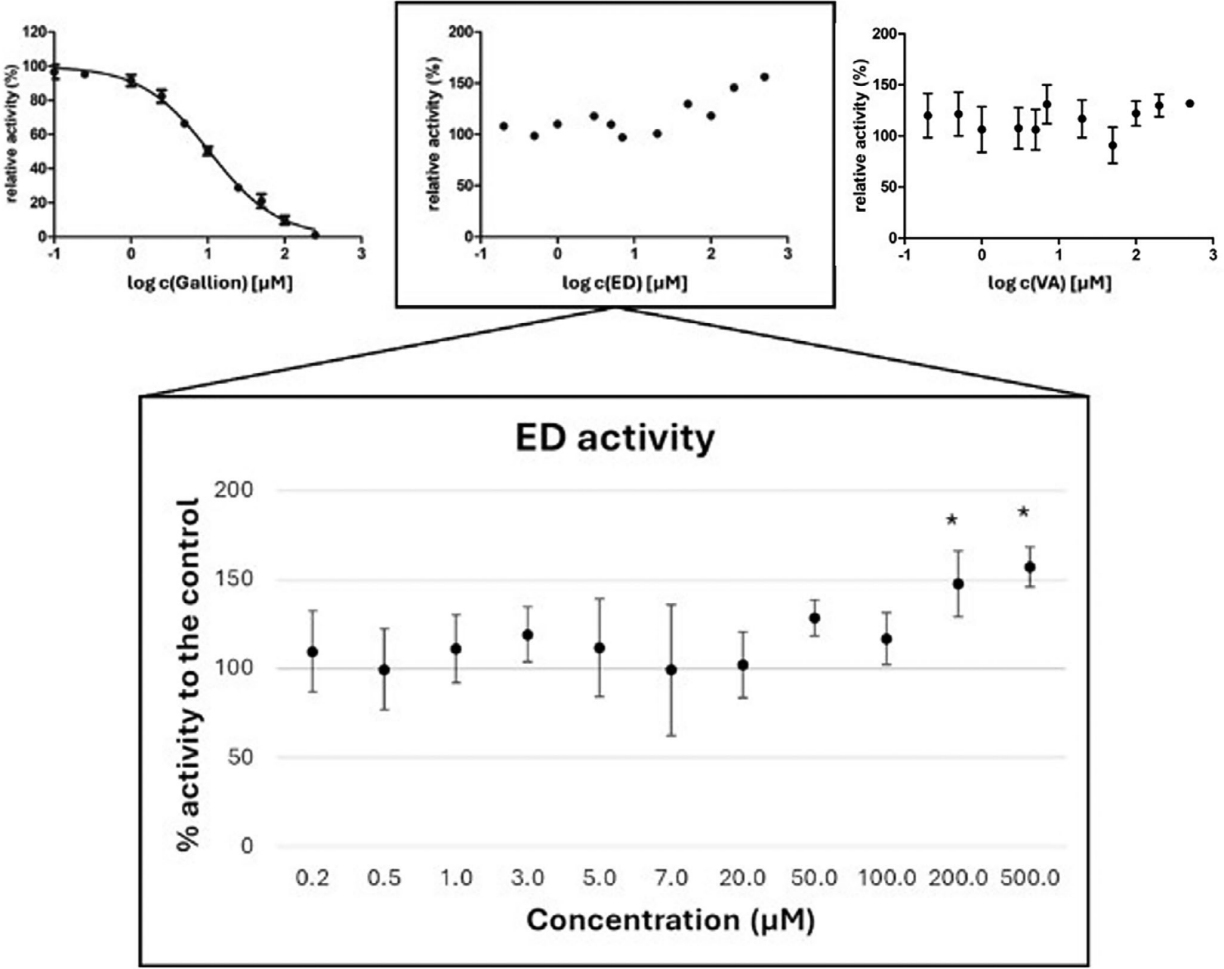
Graphs reporting the relative activity of CS with gallion, ED, and VA, at different concentrations. The log scale ranges from −1 (0.1 µm) to 3 (500 µm). On the bottom, focus on the ED activity with each datapoint represented as mean ± SEM. As reported in the graph, the activity of CS is significantly enhanced (*p* < 0.05) at 200 and 500 µm, indicated with stars above the respective values.

Following computational analysis, in vitro activity assays were performed to evaluate the inhibitory effects of gallion, ED, and VA (see Section 2.6). The assays were conducted using *P. brasiliensis* due to its well‐established assay, consistent with previous studies [27, 28]. The ligands were tested in different concentrations, starting from 0.1 µm, reaching 500 µm, and each measurement was done in triplicate. As shown in Figure 3, the CS inhibitor gallion acted as expected (full inhibition at the highest concentration tested), while on the other hand, VA did not show any significant inhibitory effect on the CS activity (*p* > 0.05). Surprisingly, ED, expected to be a promising inhibitor for the reasons stated above, significantly enhanced (*p* < 0.05) the activity of CS at the two highest concentrations tested (Figure 3). Indeed, the dose‐response activity observed for ED showed a significant though steady effect at 200 and 500 µm concentrations, suggesting that the maximal ceiling effect was plausibly reached and/or saturation was approached. While EC50 determination would be relevant for further investigations aimed at finely characterizing modulators, the saturation profile clearly states the enhancing potential of ED on CS activity. This behavior, unexpected based on the computational analysis, though interesting, could be due to several reasons, which deserve dedicated investigations as reported below.

### 3.4 ED as a Chorismate Synthase Enhancer

The serendipitous discovery of ED acting as an enhancer of CS, even if it was not expected based on the computational outcomes, was deeply investigated.

First, the computational analysis was performed on the CJ CS while the in vitro activity assay was done on the *P. brasiliensis* (Pb) orthologue due to technical reasons, like the high production yield and consolidated production protocol and optimized assay conditions (see Section 2.6). Despite the similarity of these enzymes, their active sites and overall folding are quite conserved among the bacterial kingdom; some local differences impacting ligand recruitment cannot be excluded. Moreover, peptides are intrinsically very flexible – considerably more flexible than both the inhibitor and substrate tested here. The higher flexibility might have had a role either in preventing the peptide from accessing the binding site or from arranging at the catalytic site, as described by the in silico outcome. In this respect, chemical modifications on the peptide scaffold aimed at reducing its flexibility (e.g., exploiting staple peptides) are worthy of future dedicated investigation as they might result both in proper recruitment and arrangement at the catalytic site. Nevertheless, further computational analysis was conducted on the Pb CS structure to investigate the possible mechanisms underpinning the enhancement of the CS activity observed for ED. Specifically, the existence of ancillary interaction sites other than the substrate's one, e.g., impacting protein stability and/or substrate/product turnover, was hypothesized. Of note, prior to exploring alternative surface grooves, the active site was thoroughly investigated and determined to be too sterically hindered to simultaneously accommodate three ligands, i.e., EPSP, ED, and FMN. This aligns with the structural data available so far, which shows no complexes with more than two bound ligands (last Protein Data Bank access 26th of June, 2025). Therefore, we investigated the ED capability of establishing surface interactions with CS stabilizing the protein, possibly improving its activity, and/or modulating the binding site accessibility. As a matter of fact, this might cause improved substrate/product turnover, eventually enhancing enzymatic activity.

**FIGURE 4:**
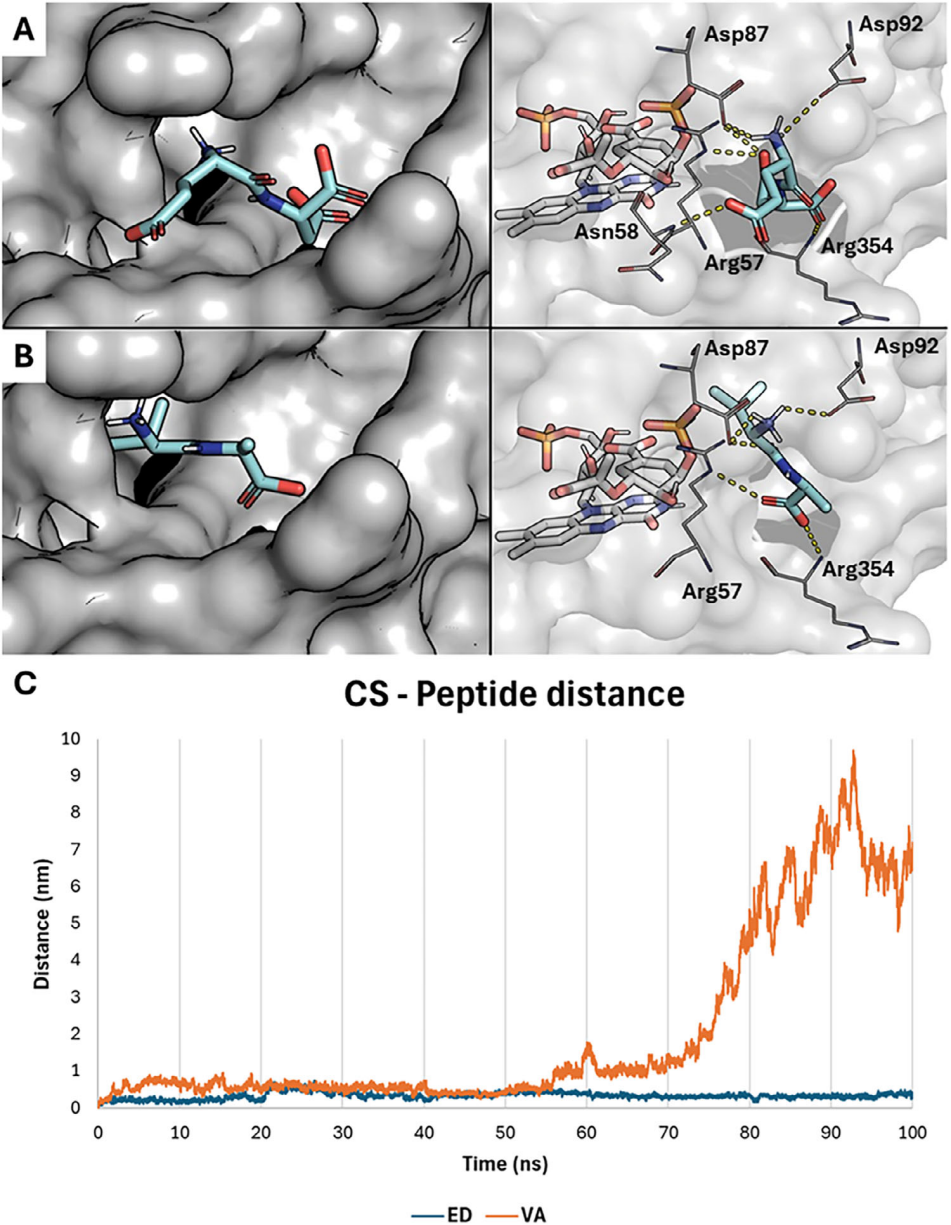
A) On the left, focus on the surface groove on Pb CS with ED docked therein represented as cyan sticks. On the right, focus on ED's interaction within the groove with a transparent surface, interacting residues reported as white lines, EPSP and FMN as white sticks, and polar interactions as yellow dashed lines. (B) On the left, focus on the surface's groove on Pb CS with VA docked therein, represented as cyan sticks. On the right, focus on VA's interaction within the groove with transparent surface, interacting residues reported as white lines, EPSP and FMN as white sticks, and polar interactions as yellow dashed lines. (C) Graph reporting the distance between the protein and the dipeptides (ED in blue, VA in orange) showing a steady interaction for ED, while a detachment for VA.

**FIGURE 5:**
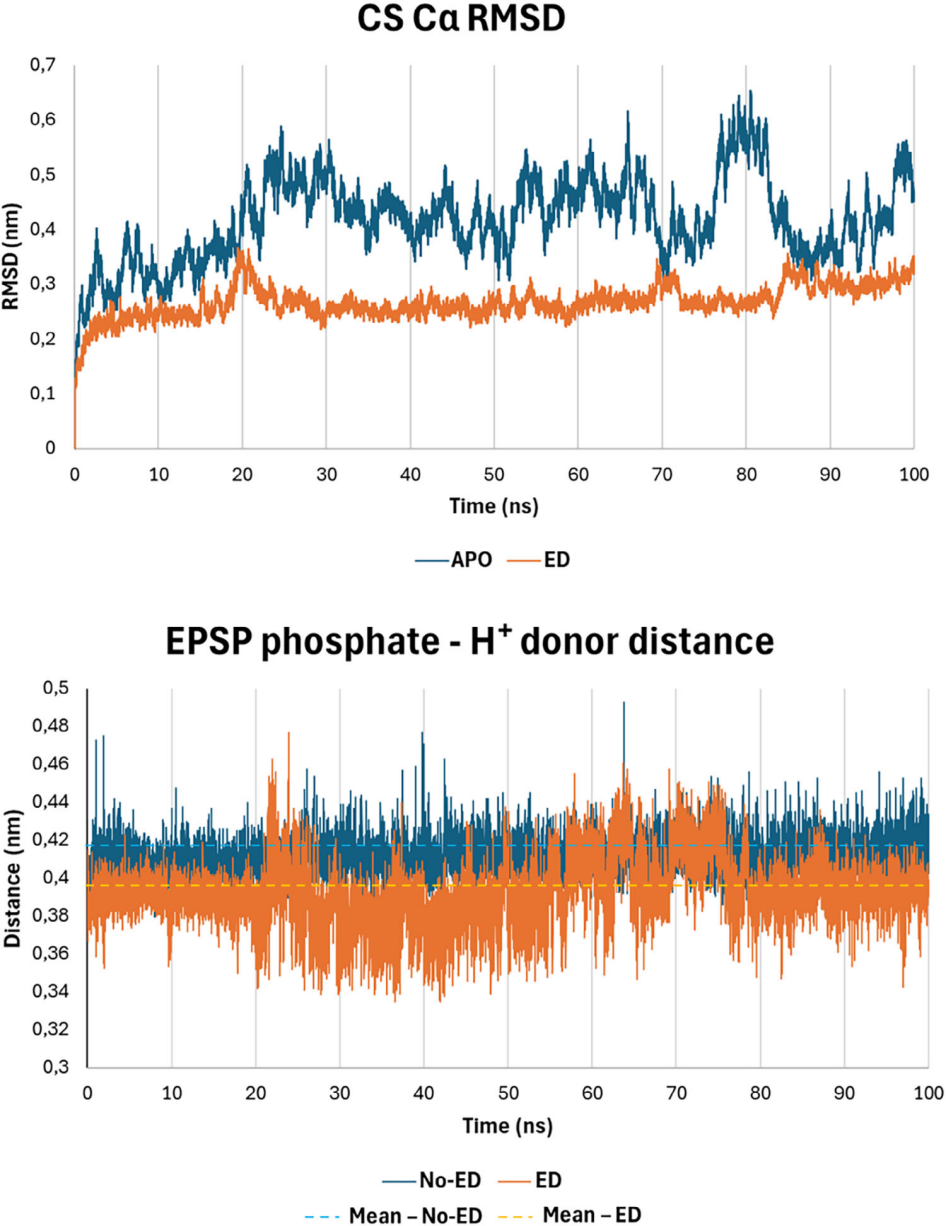
On the top, a graph representing the stability over the 100 ns MD simulation of the Pb CS – FMN – EPSP complex without (APO, blue) and with ED (ED, orange). The configuration with ED is noticeably more stable. On the bottom, graph representing the minimum distance over the 100 ns MD simulation of the EPSP phosphate with respect to possible H^+^ donors (two histidine residues and one arginine residue) when Pb CS is without (No‐ED, in blue; average equal to 0.41 nm represented as a dashed light‐blue line) and with ED (ED, in orange; average equal to 0.39 nm represented as a yellow dashed line).

After an expert visual investigation, a promising groove rich in basic residues located adjacent to the CS binding site – possibly enabling stable polar interactions of ED through its carboxylates – was identified on the protein surface (Figure 4). We docked ED and VA therein (Figure 4A,B) and proceeded with 100 ns MD simulations of Pb CS complexed alternatively with EPSP‐FMN, EPSP‐FMN‐ED, and EPSP‐FMN‐VA. The initial architecture of binding of the two peptides, despite their markedly different chemical nature, was partially comparable as they oriented their N‐ and C‐termini in the same pocket's regions (Figure S4). However, during the MD simulation, VA detached from the groove, showing a not favorable interaction, in agreement with the lack of activity observed in vitro, while ED steadily interacted there over the course of the whole simulation (Figure 4C). Comparing the stability of the complexes in the presence or absence of ED, we noticed that CS was clearly more stable in the presence of the ED, both in terms of Cα RMSD and radius of gyration (Figure 5; Figure S5). This could suggest that ED may contribute to protein folding stability, which is important for enzyme activity, as per previous studies [51]. Moreover, an interesting ED‐dependent behavior was noticed regarding the accessibility to the CS binding site and the surrounding residues of EPSP. Specifically, in the presence of ED, the binding site appears to “breath” during the simulation by opening and closing a channel to the catalytic site, which may act as an additional entry/exit channel for the substrate/product, possibly promoting their turnover (Figure S6). Additionally, it is known that CS catalyzes EPSP conversion into chorismate by removing its phosphate group, involving a proton (H^+^) transfer. The proton donor has been proposed to be a conserved histidine residue [52], but it cannot be excluded that other residues may act as Brønsted‐Lowry acids (i.e., arginine), as previously described [53]. For this reason, we monitored the minimum distance kept between the side chains of the three residues of CS theoretically able to act as Brønsted‐Lowry acids (His17, Arg56, and Arg319; indexing as per our model) surrounding the EPSP phosphate group. As shown in Figure 5, the distances remained lower in the presence of ED rather than in its absence. This could point to the possible capability of ED to promote the substrate/product turnover, also facilitating the proton transfer by maintaining the phosphate group closer to potential proton donors.

Of note, CS is not known to have any activators, and, like other flavin‐dependent enzymes, its activity mainly depends on the substrate availability and co‐factor recycling [21, 54]. Nevertheless, other enzymes of the shikimate pathway, like chorismate mutase and 3‐deoxy‐D‐arabino‐heptulosonate 7‐phosphate (DAHP) synthase, have documented peptide modulators [55]. Interestingly, the proposed mechanism aligned with the evidence that substrate availability is a limiting factor for the CS reaction, as it highlights the ability of ED to regulate accessibility to the catalytic site and optimize the surroundings of FMN to facilitate the reaction. Last, we compared the residues shaping the identified external groove in Pb CS with the corresponding residues in CJ CS (Figure S6). Looking at the primary sequence, there is a 50% identity and 60% similarity out of the selected 10 residues. However, the locally computed RMSD is 1.308 Å, highlighting an overall structural similarity and suggesting a similar behavior when such a groove can be suitably drugged by specifically designed peptides. These findings convincingly point to the likelihood of the proposed mechanism underlining the need for further dedicated investigations to assess the relevance of the proposed CS modulation in a real‐world scenario.

## 4 Conclusion

To conclude, ED was identified in vitro as a CS enhancer, rather than an inhibitor, as per in silico analysis. However, the chemical basis of this conflictual outcome was deeply analyzed in silico. Among the possible explanations, we could propose the incapability of ED to reach the inner part of the Pb CS catalytic site being “trapped” within a surface groove – possibly due to ED's intrinsic high flexibility – and likely influencing substrate/product turnover by stabilizing the enzyme structure and promoting better access to the catalytic site. Therefore, future work should explore the potential of peptidomimetic alternatives with reduced flexibility to more closely resemble the natural substrate.

While our study demonstrated the enhancing activity capacity of ED for Pb CS, it is reasonable to expect that similar effects may occur in other bacteria relevant to food safety and production, including *C. jejuni*. This is particularly significant considering that the shikimate pathway is highly conserved among bacterial species. The transferability of our findings to *C. jejuni* and other foodborne or environmental spread pathogens warrants dedicated investigation, as it could have profound implications for controlling bacterial populations in food matrices.

Crucially, this study underscores the possible significance of matrix effects on bacterial growth, as certain peptides might influence bacterial fitness in protein‐rich substrates when enhancing CS activity. Overall, this study provides a solid blueprint for further dedicated analysis of CS, broadening the knowledge regarding the chemical characteristics of its binding site and its general activity. The serendipitous discovery of ED as a CS enhancer (rather than an inhibitor), and the precise descriptions of the underpinning mechanisms, open new research avenues and give clues on a possible scaffold to be used to develop effective and peptide‐based CS modulators for a more precise control of bacterial growth in diverse food matrices.

## Author Contributions

**Lorenzo Pedroni**: Methodology, Formal analysis, Investigation, Writing – original draft, Writing – review & editing; **Katharina Fuchs**: Methodology, Investigation, Formal Analysis; **Gianni Galaverna**: Writing – review & editing, Conceptualization; **Peter Macheroux**: Conceptualization, Methodology, Supervision; **Luca Dellafiora**: Investigation, Writing – original draft, Writing – review & editing, Conceptualization, Supervision.

## Conflicts of Interest

The authors declare no conflicts of interest.

## Supporting information




**Supporting File**: gch270063‐sup‐0001‐SuppMat.docx

## Data Availability

The raw data generated in this work will be made available upon request to the corresponding author.
