# Supplementary material for: Integrated In Silico – In Vitro Study Investigating Dipeptides as Chorismate Synthase Modulators: Spotlight on Its Mechanism of Action
Source: Glob Chall. 2025 Oct 30;9(12):e00316. doi: 10.1002/gch2.202500316 (PMC12697075; doi:10.1002/gch2.202500316)
Supplement: Supplementary file 1 — Supporting File: gch270063‐sup‐0001‐SuppMat.docx [file GCH2-9-e00316-s001.docx]

Supporting Material

**Integrated *in silico* – *in vitro* study investigating dipeptides as chorismate synthase modulators: spotlight on its mechanism of action**

Lorenzo Pedroni^1,#^, Katharina Fuchs^2,#^, Gianni Galaverna ^1^, Peter Macheroux^2^, Luca Dellafiora^1,*^

^1^ Department of Food and Drug, University of Parma, Parma, Italy

^2^ Institute of Biochemistry, Graz University of Technology, Petersgasse 12/II

^#^ These authors contributed equally to the work

* Corresponding author: Luca Dellafiora, Department of Food and Drug, University of Parma, Parco Area delle Scienze 27/A, 43124 Parma, Italy. Phone: +39 0521 906070. Email: [luca.dellafiora@unipr.it](mailto:luca.dellafiora@unipr.it)

**Co-authors e-mail list:** Lorenzo Pedroni, lorenzo.pedroni@unipr.it; Katharina Fuchs, [katharina.fuchs@tugraz.at](mailto:katharina.fuchs@tugraz.at); Gianni Galaverna, [gianni.galaverna@unipr.it](mailto:gianni.galaverna@unipr.it); Peter Macheroux,  [peter.macheroux@tugraz.at](mailto:peter.macheroux@tugraz.at)

**Data Availability Statement:** The raw data generated in this work will be made available upon request to the corresponding author.

**Funding Statement:** Nothing to disclose

**Conflict of Interest Disclosure:** The authors have nothing to disclose


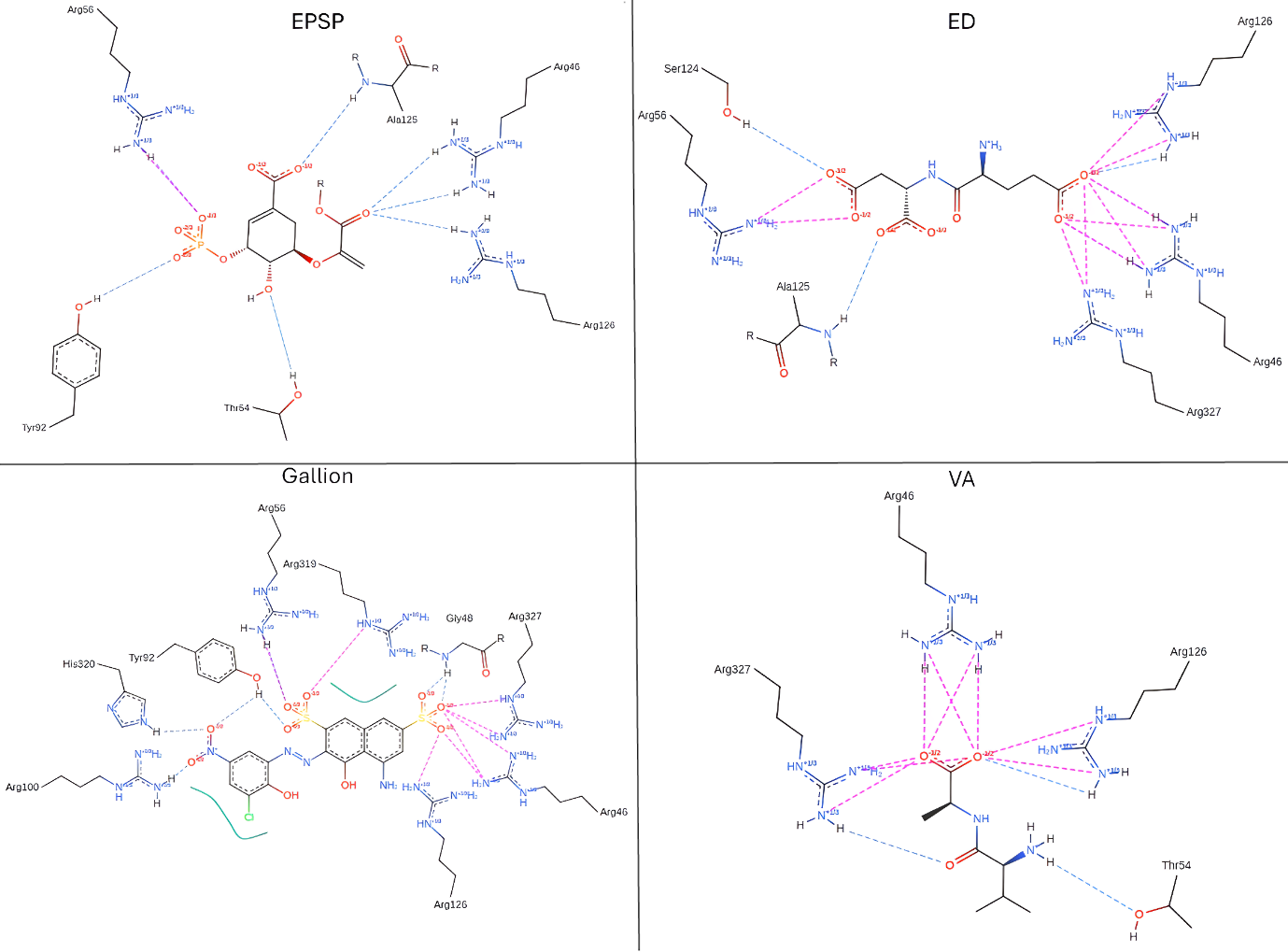


**Figure** **S1**. 2D representations of the interaction patterns between the ligands EPSP, ED, Gallion, and VA and the residues within the CS binding pocket. Hydrogen bonds are shown as dashed blue lines, while salt bridges are as dashed magenta lines. The figures have been generated using PoseEdit web server by uploading the calculated binding architectures (https://proteins.plus).


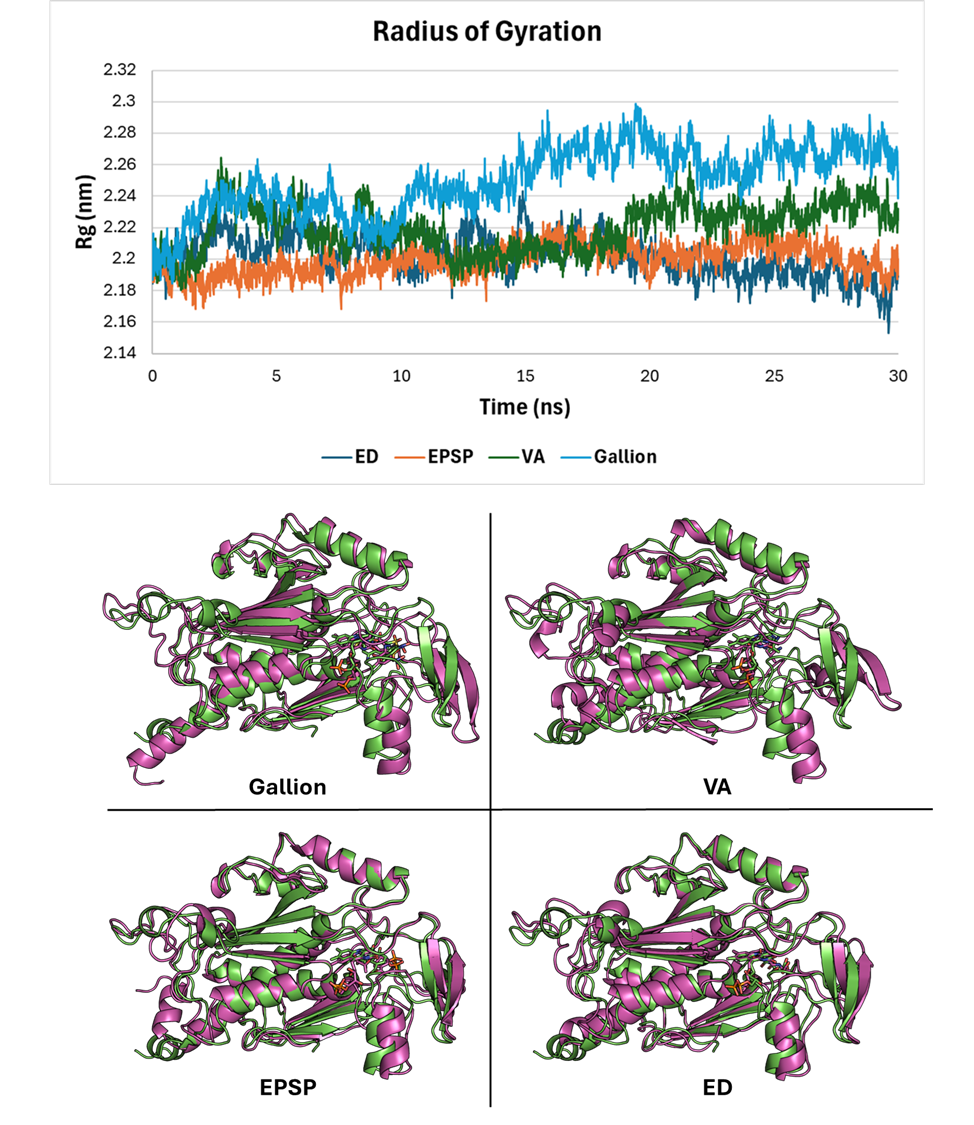


**Figure** **S2**. On the top, radius of gyration (Rg) for the MD simulations of ED, EPSP, VA, and Gallion. ED and EPSP exhibit similar Rg trends, indicating comparable structural compactness and stability throughout the simulations. In contrast, VA and Gallion show higher Rg values, suggesting a more expanded or flexible conformation during the MD simulations. On the bottom, superimposition between the first (green) and last (magenta) frame from each MD simulation. The corresponding RMSD values are 2.71 Å for gallion, 3.89 Å for VA, 1.69 Å for EPSP, and 1.99 Å for ED.


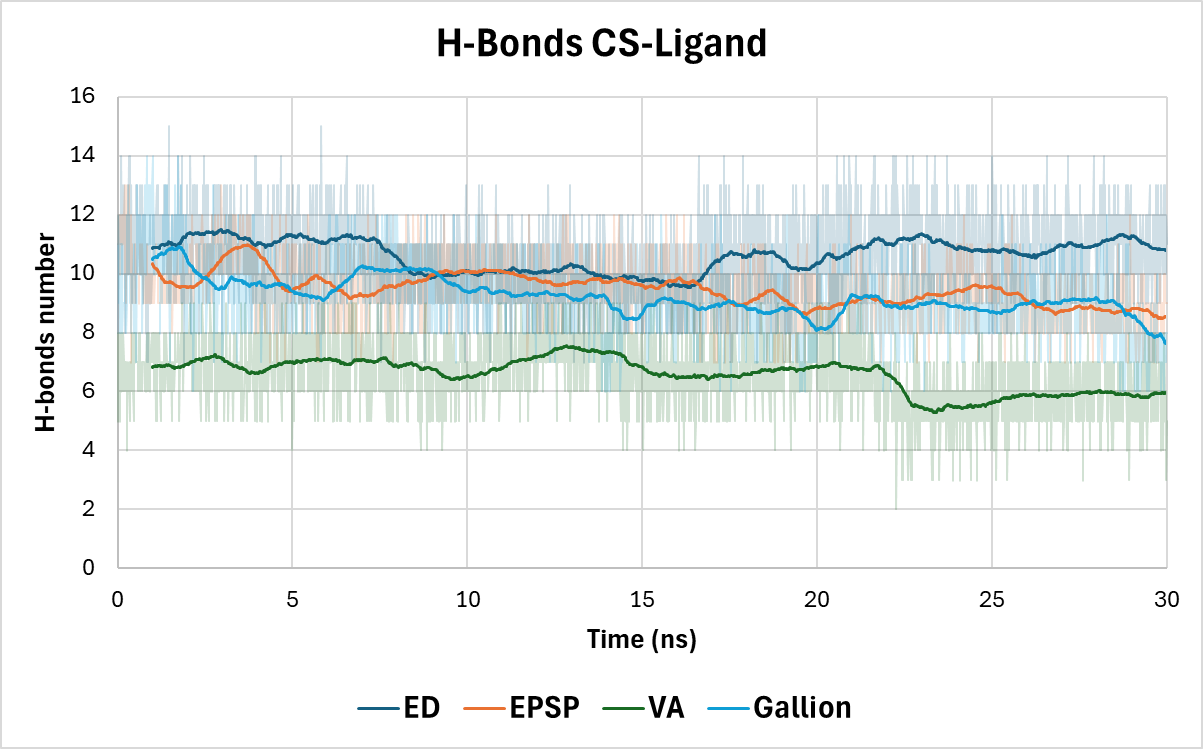
**Figure S3**. Hydrogen bonds analysis between each ligand - ED, VA, EPSP, and Gallion - and CS during MD simulations. Transparent lines represent raw data, while the solid lines in the foreground show the moving average, providing a clear visualization of the overall trends.


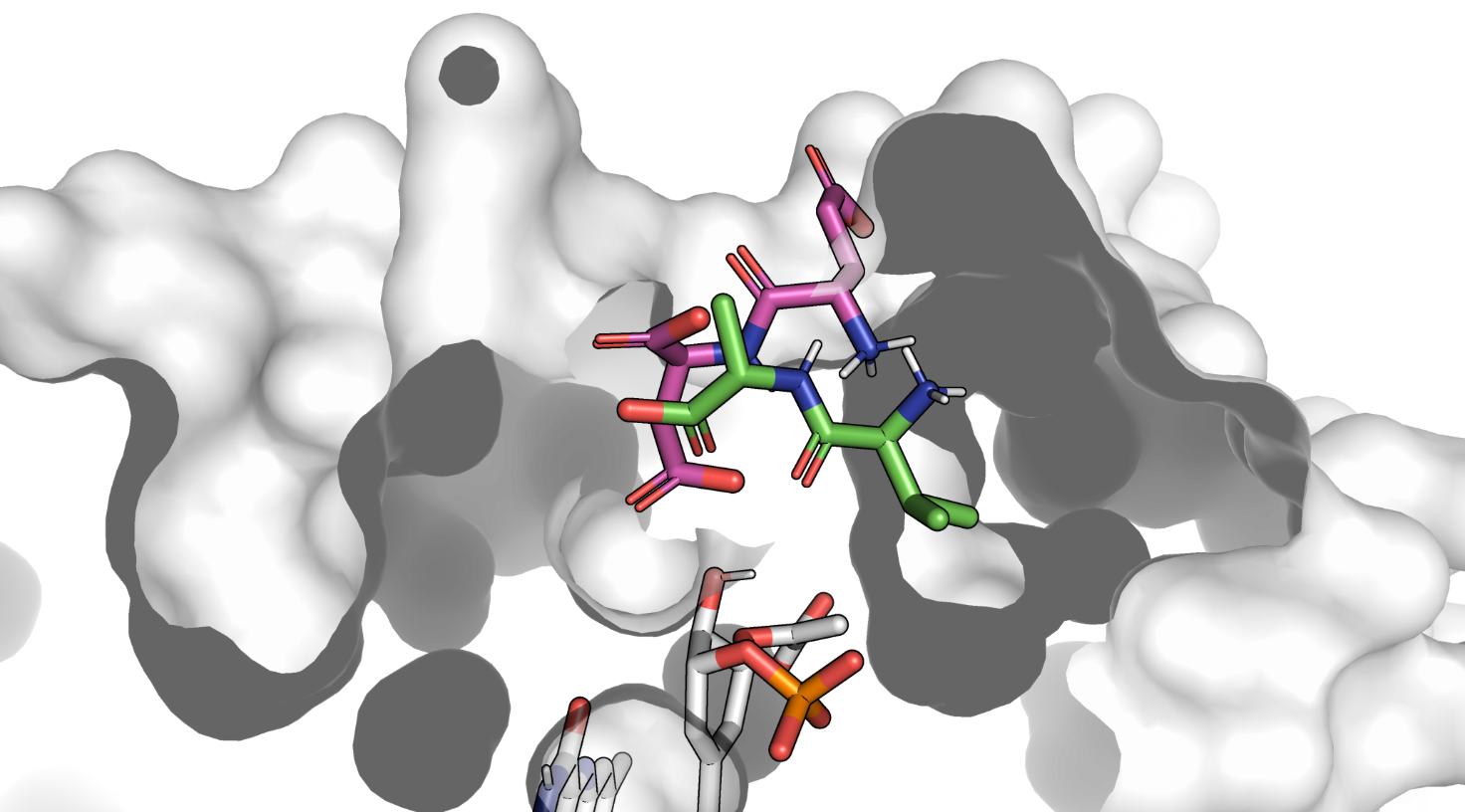


**Figure S4**. Superimposition of the docking poses of ED (magenta sticks) and VA (green sticks) within the identified external groove. The protein is shown as a white surface. At the bottom, portions of EPSP and FMN are shown as white sticks.


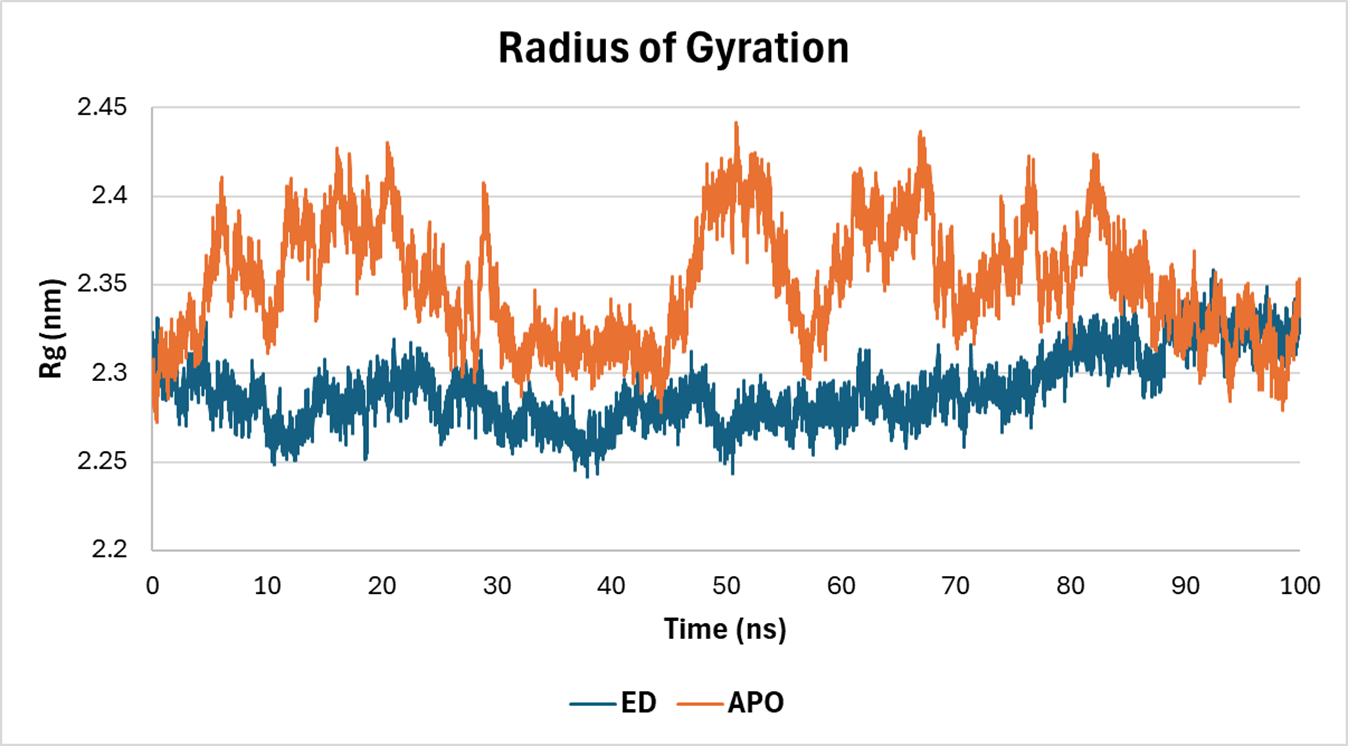


**Figure S5.** Radius of gyration (Rg) for CS complexed with EPSP alone (APO) and with EPSP plus ED docked in the identified off-site region (ED). The presence of ED results in a consistently lower Rg, indicating a more compact and stable protein conformation compared to the system without ED.


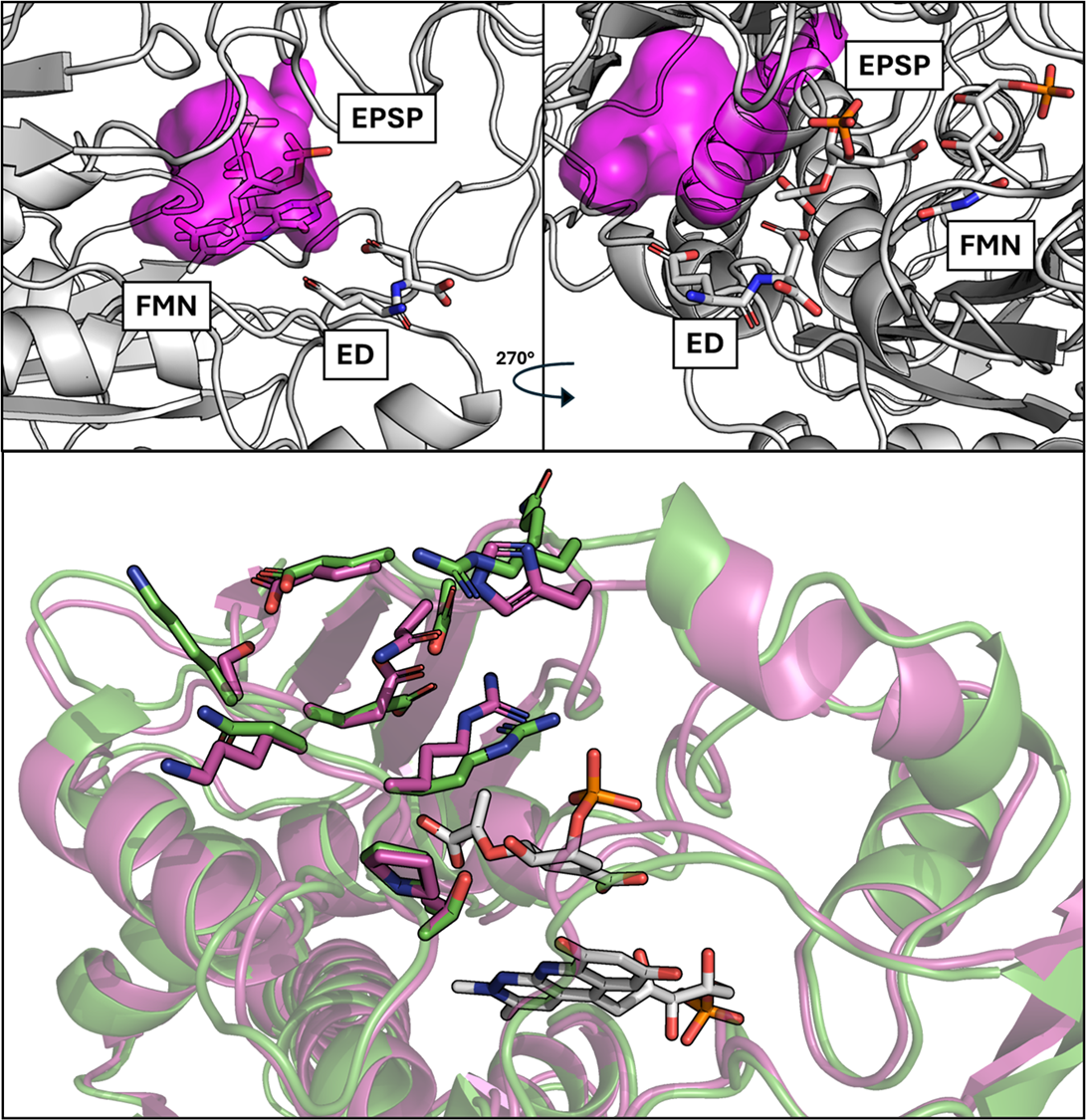


**Figure S6.** On the top, snapshot from two different orientations of Pb CS (shown as white cartoon) in complex with FMN, EPSP, and ED (shown as white sticks) bound within the identified external groove. The channel formed is represented as a magenta surface. On the bottom, comparison of the residues shaping the external groove in *P. brasiliensis* (namely, TPRNEK---EDQR, shown as green sticks) with the corresponding residues in *C. jejuni* (namely, TPRKES---ENAH, shown as magenta sticks). FMN and EPSP are shown as white sticks.

| **Table** **S1**. Docking scores of the top 40 dipeptides – i.e. the top 10% – alongside Gallion (a known inhibitor) and EPSP (the natural substrate). | | | |
| --- | --- | --- | --- |
| **Ligand** | **PLP Score** | **Ligand** | **PLP Score** |
| tyrglu | 94.81 | argasp | 85.07 |
| tyrhis | 93.97 | hisglu | 84.77 |
| gluglu | 92.17 | argglu | 84.67 |
| trphis | 91.41 | **gluasp** | **84.44** |
| glulys | 91.28 | gluile | 83.96 |
| glugln | 90.98 | trpcys | 83.72 |
| tyrasp | 90.95 | asplys | 83.70 |
| glutyr | 89.55 | aspglu | 83.69 |
| pheasp | 88.29 | hisasp | 83.57 |
| gluarg | 88.16 | lysmet | 83.52 |
| tyrtyr | 88.02 | tyrmet | 82.80 |
| trpasp | 87.87 | trpmet | 82.74 |
| gluasn | 87.81 | glnasp | 82.69 |
| phehis | 87.35 | aspasp | 82.53 |
| gluleu | 86.69 | tyrgln | 82.36 |
| asptyr | 86.38 | lystyr | 82.21 |
| trpasn | 86.00 | asnglu | 82.09 |
| asptrp | 85.92 | glumet | 81.86 |
| glnglu | 85.91 | **EPSP** | 70.03 |
| pheglu | 85.79 | **valala** | 58.29 |
| trpglu | 85.75 | **Gallion** | 43.75 |
| lysasp | 85.14 |  |  |
| *Note*. The ligands tested with further MD simulations and the activity *in* *vitro* assays are highlighted in bold font. | | | |
